# PoseNeRF: In Situ 3D Reconstruction Method Based on Joint Optimization of Pose and Neural Radiation Field for Smooth and Weakly Textured Aeroengine Blade

**DOI:** 10.3390/s25196145

**Published:** 2025-10-04

**Authors:** Yao Xiao, Xin Wu, Yizhen Yin, Yu Cai, Yuanhan Hou

**Affiliations:** 1 National Key Laboratory of Aerospace Power System and Plasma Technology, Air Force Engineering University, Xi’an 710038, China; xiaoy_airforce@126.com (Y.X.); wuxin18892059516@163.com (X.W.); m13201659925@163.com (Y.H.); 2 Institute of Aeronautics Engine, School of Mechanical Engineering, Xi’an Jiaotong University, Xi’an 710049, China; yinyizhen0@163.com

**Keywords:** aeroengine blades, 3D reconstruction, deep learning, neural radiance field, industrial borescope

## Abstract

Digital twins are essential for the real-time health management and monitoring of aeroengines, and the in situ three-dimensional (3D) reconstruction technology of key components of aeroengines is an important support for the construction of a digital twin model. In this paper, an in situ high-fidelity 3D reconstruction method, named PoseNeRF, for aeroengine blades based on the joint optimization of pose and neural radiance field (NeRF), is proposed. An aeroengine blades background filtering network based on complex network theory (ComBFNet) is designed to filter out the useless background information contained in the two-dimensional (2D) images and improve the fidelity of the 3D reconstruction of blades, and the mean intersection over union (*mIoU*) of the network reaches 95.5%. The joint optimization loss function, including photometric loss, depth loss, and point cloud loss is proposed. The method solves the problems of excessive blurring and aliasing artifacts, caused by factors such as smooth blade surface and weak texture information in 3D reconstruction, as well as the cumulative error problem caused by camera pose pre-estimation. The *PSNR*, *SSIM*, and *LPIPS* of the 3D reconstruction model proposed in this paper reach 25.59, 0.719, and 0.239, respectively, which are superior to other general models.

## 1. Introduction

With the introduction of the digital twin concept, researchers hope to construct a digital twin model to better manage and monitor the health status of aeroengines. The foundation for constructing an aeroengine digital twin model is to reconstruct the true three-dimensional (3D) model of the key components of aeroengines to reflect changes in their structural characteristics [1]. As a key component of aeroengines, blades compress and expand the gas through high-speed rotation, generating strong power to propel the aircraft forward [2,3]. Blades work under harsh conditions such as high temperature, high pressure, high load, rotation, and strong corrosion. When impacted by foreign objects, the blades are extremely prone to structural damage, seriously affecting the aeroengine’s performance and flight safety [4,5]. Therefore, the research into in situ 3D reconstruction technology of aeroengine blades is of great significance.

Currently, the binocular stereo vision principle [6,7] has been utilized in borescopes to achieve the in situ measurement and 3D reconstruction of aeroengine blades. However, due to the influence of smooth blade surface, weak texture information, complex light inside aeroengines, and using only a single frame image for reconstruction, the fidelity of the aeroengine blades 3D model reconstructed by binocular borescope is poor. High-quality reverse 3D models of aeroengine blades are mostly obtained by ex situ 3D reconstruction methods, mainly including contact reconstruction and contactless reconstruction [8,9,10,11]. Ex situ 3D reconstruction requires blade disassembly under laboratory conditions. The ex situ methods are complex and inefficient and increase the difficulty of blade 3D reconstruction. These limitations make it impossible for frontline inspectors to obtain the structural status information of blades in the out-field in the first instance, thus making it impossible to evaluate the aeroengine status in the first instance.

Mildenhall et al. [12] combined classical volume rendering with neural network and pioneered the use of neural radiance field (NeRF) for 3D reconstruction. Unlike discrete explicit 3D representation such as point clouds, voxels, meshes, etc., NeRF is a continuous high-fidelity implicit model. This method is a monocular 3D reconstruction method without the introduction of binoculars, structured light, line laser, or other auxiliary hardware. The NeRF-based method provides a theoretical basis for in situ high-fidelity 3D reconstruction of aeroengine blades. In this paper, an in situ high-fidelity 3D reconstruction method for aeroengine blades is proposed. In the reconstruction method, a monocular borescope is utilized as the fore-end to collect data and NeRF is utilized as the back-end to reconstruct the blades.

However, most NeRF methods [13,14,15,16,17,18] are designed for a general dataset. These methods struggle to deal with such challenges as smooth blade surface, weak texture information, complex light inside the aeroengine, etc., and do not consider the relationship between sequential frame images. These methods need accurate camera pose information, which is difficult to obtain via borescope. Therefore, the existing NeRF methods cannot be directly used for the in situ 3D reconstruction of aeroengine blades. To solve the above problems, we adopt an end-to-end NeRF method based on cone sampling for the in situ 3D reconstruction of aeroengine blades. Our method takes full account of the relationship between sequential frame images and can compute the camera pose synchronously during the training process, avoiding the cumulative error of 3D reconstruction caused by camera pose pre-estimation.

In this paper, an in situ high-fidelity 3D reconstruction method for aeroengine blades based on implicit functions is proposed. This method uses a monocular borescope to collect blades images in situ and then uses a deep learning method based on complex network theory to filter out background noise. Finally, the end-to-end NeRF method proposed in this paper specifically for aeroengine blades is utilized to achieve the in situ 3D reconstruction of the blades. We carry out experiments to verify the performance of the background filtering method and 3D reconstruction method designed in this paper. The results show that the mean intersection over union (mIoU) of our background filtering method reaches 95.7%, and the peak signal-to-noise ratio (PSNR) [19] of our 3D reconstruction method reaches 25.61, which are better than the results of other general methods. Ablation experiments are also conducted to study the influence of background filtering, cone sampling and joint optimization loss function on the 3D reconstruction method. The experimental results show that cone sampling has the greatest effect on improving the 3D reconstruction of the blades, and the joint optimization loss function mainly improves the fidelity of the 3D reconstruction of the blades by affecting the optimization of camera pose.

Our contributions are mainly as follows:

(1) An in situ high-fidelity 3D reconstruction method, named PoseNeRF, for aeroengine blades, based on the joint optimization of pose and NeRF is proposed. The method is of great significance for aeroengine out-field detection, reverse reconstruction, digital twinning, and life assessment.
(2) A deep learning method of background filtering based on complex network theory (ComBFNet) is designed. The method improves the background filtering effect by improving the feature extraction ability of the backbone. Background filtering can improve the fidelity of blade 3D reconstruction.
(3) An implicit 3D reconstruction method based on cone sampling and “pose–NeRF” joint optimization is designed to solve the problems of excessive blur, aliasing artifacts, and other problems caused by smooth blade surface, weak texture information, and other factors, as well as the cumulative error caused by camera pose pre-estimation.

## 2. Related Works

### 2.1. Image-Based 3D Reconstruction

**Multi-view stereo:** Image-based 3D reconstruction restores the 3D structure of an object from multiple two-dimensional (2D) images, renders it, and finally performs the virtual expression of the objective world in the computer. Most of the traditional 3D reconstruction methods first reconstruct the sparse point cloud of the target through structure from motion (SFM) [20,21], and unify different camera perspectives into the same coordinate system to obtain the camera pose parameters and solve the size of the reconstructed object. Then, according to the obtained camera pose parameters, dense point cloud reconstruction is performed through multi-view stereo (MVS) [22]. Then, the point cloud is transformed into mesh by surface reconstruction, and texture reconstruction and visual rendering are carried out. SFM mainly includes incremental SFM [23,24] and global SFM [25,26,27]. Among them, incremental SFM is widely used because of its high robustness. MVS mainly includes depth map fusion-based methods [22] and spatial patch diffusion-based methods [28,29]. In recent years, with the development of deep learning, Yao et al. [30] proposed MVSNet, which pioneered the application of deep learning methods to MVS. Subsequently, improved algorithms such as Point MVSNet [31], Cascade MVSNet [32], PatchmatchNet [33], and TransMVSNet [34] were proposed one after another. The above methods are all based on the principle of photometric consistency. The target to be reconstructed is simplified as an object that conforms to Lambertian effects. It is assumed that when the target is observed from different angles, the color of the same point on the target surface is consistent, and the target is represented in a discrete way. However, the actual situation is that due to the influence of light and material, the same point on the target surface shows different colors under different angles, presenting as non-Lambertian effects, and the real space is continuous rather than discrete. This directly limits the accuracy of the MVS based methods in principle, resulting in problems such as holes, texture aliasing, and loss of details in the final reconstructed model.

**Neural radiance field:** The proposal of NeRF [12] innovatively changed the 3D representation of scene, transforming the traditional discrete explicit representation into continuous implicit representation. By introducing the concept of volume rendering, NeRF constructs a continuous radiance field function to implicitly represent the scene, and believes that the color of the target surface is not only related to the position, but also related to the viewing angle, which solves the problem of non-Lambertian effects. Subsequently, NVIDIA proposed Instant-NGP to solve the problem of the slow running-speed of NeRF. It uses hash encoding to encode the spatial position, which improves the running speed of NeRF by several orders of magnitude [13]. Wang et al. [16] proposed a new neural surface reconstruction method called NeuS, which used a zero-level set of signed distance function (SDF) to represent a surface and designed a new volume rendering method to train neural SDF descriptions, significantly improving the quality of 3D surface reconstruction. Xu et al. [17] proposed Point-NeRF by combining NeRF with deep MVS, which used a neural 3D point cloud with neural features to establish an implicit radiance field model. Point-NeRF can be rendered effectively by aggregating neural point features near scene surfaces, in a ray-marching-based rendering pipeline, which improves the fidelity and speed of 3D reconstruction. However, the above NeRF method assumes that only one ray passes through each pixel in the image during the sampling process, which will cause excessive blur and aliasing artifacts when reconstructing the aeroengine blades with smooth surfaces and weak texture information. In addition, the above methods need to input the known camera pose information. In the actual scene, the camera pose is usually estimated in advance by incremental SFM. However, the incremental SFM will fail when the camera poses change greatly. Due to the limitations of the engine probe hole and the complex internal structure, it is often difficult to precisely control the position and pose of the borescope. Therefore, incremental SFM is not suitable for the in situ 3D reconstruction of aeroengine blades. Moreover, the above method completely separates the camera pose estimation and 3D reconstruction into two independent parts, which means that the camera pose estimation error brings an uncontrollable superposition effect to 3D reconstruction. To solve the above problems, an end-to-end NeRF method for the in situ 3D reconstruction of aeroengine blades is designed, which uses cone sampling instead of single ray sampling, fully considers the relationship between sequential frame images, and jointly optimizes camera pose and NeRF in training.

### 2.2. Three-Dimensional Reconstruction of Aeroengine Blades

The 3D reconstruction of aeroengine blades is a key technology in the fields of manufacturing, damage detection, additive repair, digital twinning and so on. Therefore, researchers have carried out research on the 3D reconstruction of blades. Li et al. [8] used a six-axis mechanical arm combined with line-laser scanning to obtain the point cloud of blades, and then applied a moving least-squares surface to generate geometric deviation. The geometric deviation was utilized to subdivide and cluster the point cloud, achieving the 3D reconstruction of blades. Mineo et al. [35] proposed a new boundary point detection method and a spatial FFT-based filtering method to the detect sharp edges and corner points of aeroengine blades, achieving the edge reconstruction of blades. Jing et al. [10] proposed a method for remanufacturing blade weld-seam recognition and model reconstruction based on a self-developed binocular vision system. Firstly, the computational cost of 3D reconstruction and the complexity of point cloud processing were reduced through coarse positioning. Then, the precise positioning of the weld seam was achieved through point cloud segmentation based on a region growth algorithm and a point cloud normal filtering algorithm, resulting in a blade weld-seam model with precise boundaries. Xu et al. [36] proposed a binocular vision 3D reconstruction method based on enhanced features. Firstly, circular markings are pasted on the surface of blades to enhance their surface features. Then, a binocular camera is used to capture the blade from multiple angles, and the center point feature-matching method is used to match the corresponding marked points in the left and right images. Finally, the point cloud of blades is computed by the principle of binocular vision 3D reconstruction. Song et al. [37] proposed a 3D reconstruction method for blade profiles based on laser point cloud data. Laser displacement sensors were used to scan and sample the blade surface from multiple angles, and then mathematical modeling principles from point to line to surface were used to construct an accurate and smooth blades surface. Although the above research has achieved 3D reconstruction of aeroengine blades, these methods all require the disassembly of aeroengine blades, which is ex situ reconstruction, a complex operation, low efficiency, and cannot be used for the in situ high-fidelity 3D reconstruction of blades in the out-field. Moreover, these methods cannot be achieved through a single camera and require other hardware such as binoculars, structured light, or line lasers to assist in data collection. Our method is an in situ 3D reconstruction method of blades, which only needs to collect data through a monocular borescope, and does not need to dismantle the blades.

## 3. Method

**Figure 1:**
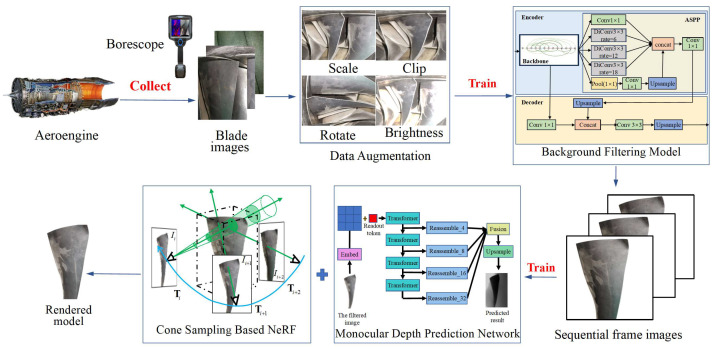
Pipeline of the in situ high-fidelity 3D reconstruction method for aeroengine blades based on joint optimization of pose and NeRF.

The pipeline of our PoseNeRF is shown in Figure 1. Firstly, the sequential frame images of blades are collected via monocular borescope and the dataset is expanded by data augmentation methods. Then, the useless background information in the 2D images is filtered by the deep learning network designed based on complex network theory. Finally, the filtered sequential frame images are input into the monocular depth prediction network and the cone sampling based NeRF for joint optimization to complete the in situ high-fidelity 3D reconstruction of aeroengine blades.

### 3.1. Background Filtering

**Figure 2:**
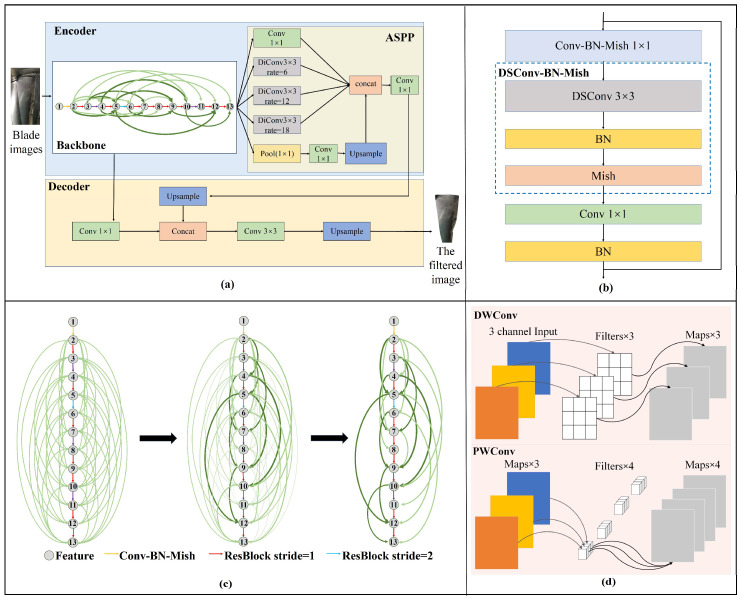
Overview of ComBFNet. (**a**) Pipeline of ComBFNet. (**b**) Architecture of ResBlock. (**c**) Construction process of ComNet. (**d**) Illustration of DSConv.

The in situ 3D reconstruction method for aeroengine blades proposed in this paper is based on 2D images, which contain a large amount of useless background information. If the images are not preprocessed and directly used for 3D reconstruction, a large number of useless 3D structures will be reconstructed. The redundant structures bring unnecessary trouble to the post-processing of the 3D data. In addition, the useless background information also affects the fidelity of the in situ 3D reconstruction of the blades. Therefore, a deep learning method is adopted to filter out the background of images collected through a monocular borescope. In this section, inspired by the parallel visual pathways model [38], ComBFNet based on complex network theory is designed [39,40,41], as shown in Figure 2. ComBFNet mainly consists of an encoder and a decoder, and the encoder is composed of a backbone and atrous spatial pyramid pooling (ASPP).

#### 3.1.1. Backbone

Research has verified that at least 35 regions in the cerebral cortex are related to visual functions [38]. The parallel visual pathways model suggests that advanced brain regions related to vision do not simply receive signals from the retina through a neural pathway alone, but rather process neural signals through multiple pathways with different lengths. As is well-known, a convolutional neural network (CNN) [42,43,44,45] is constructed in an imitation of human visual neural systems. Therefore, we argue that the CNN employed for image-feature extraction should have a similar topological structure to the parallel visual pathways model. The CNN should be a complex network between regular networks and random networks, with a small-world effect. It has a small average path length and a large clustering coefficient as shown in Equation (1). Based on the above analysis, an innovative backbone for the feature extraction of aeroengine blades based on complex network theory is designed in this paper. Where *N* is the number of network nodes, and *d_ij_* is the path length between node *i* and node *j*. Where *N* is the number of network nodes, *R_i_* is the number of triangles formed by node *i* and its neighbor nodes, and *k_i_* is the number of first-order neighbor nodes of node *i*.

(1) $$\begin{array}{c} \left\{\begin{array}{c} L=\frac{1}{\frac{1}{2}N(N-1)}\sum_{i\geq j}\;d_{ij}\\ C=\frac{1}{N}\sum_{i=1}^{N}\;\frac{2R_{i}}{k_{i}(k_{i}-1)}\; \end{array}\right. \end{array}$$

Considering real-time performance, depthwise separable convolution (DSConv) [46,47] and ordinary convolution (Conv) are utilized as the basic layers for feature extraction. DSConv consists of depthwise convolutions (DwConv) and pointwise convolutions (PwConv). To prevent gradient explosion and vanishing, an inverted residuals block (ResBlock) is constructed as the basic block for feature extraction, as shown in Figure 2b. The ResBlock consists of a Conv block (Conv-BN-Mish), which consists of a Conv, a batch normalization layer (BN), a Mish activation function, and a DSConv block (DSConv-BN-Mish), and the extracted features *F_out_* can be computed by Equation (2), where *F_in_* is the input features of ResBlock, *F_Conv_* is the features extracted by Conv-BN-Mish, *F_DSConv_* is the features extracted by DSConv-BN-Mish, *DSConv^a^_s_* is the Conv-BN-Mish with kernel size *a × a* and stride *s*, and *DSConv^a^_s_* is DSConv-BN-Mish. Firstly, a chain network with only *a* path using a Conv-BN-Mish and 13 ResBlock as edges and extracted features as nodes is constructed. The chain network performs 16× downsampling on the input image. Then, Conv-BN-Mish is used to expand the chain network into a global coupling network to provide different paths for feature extraction. Then, the global coupling network is trained to obtain the weights of each path. Paths with tiny weights are considered to have a poor feature extraction capacity in the network; therefore, they are deleted. Finally, the network is retrained to obtain a blade feature extraction backbone (ComNet) with multiple paths and a small-world effect, as shown in Figure 2d.

(2) $$\begin{array}{c} \left\{\begin{array}{l} F_{\mathrm{Conv}}=Mish\;(BN({Conv}_{1}^{1}(F_{in})))\\ F_{\mathrm{DSConv}}=Mish\;(BN({DSConv}_{1or2}^{3}(F_{\mathrm{Conv}})))\\ F_{\mathrm{out}}=BN({Conv}_{1}^{1}(F_{DSConv}))+F_{\mathrm{in}} \end{array}\right. \end{array}$$

#### 3.1.2. ASPP and Decoder

ASPP is designed as a multi-scale feature fusion module to enhance features by using dilated Conv. ASPP in parallel processes the 16× downsampling feature maps extracted from ComNet by using 1 × 1 Conv, max pooling, and dilated Conv with dilation rates of 6, 12, and 18, respectively to obtain five feature maps with different receptive fields. Then, the five feature maps are concatenated at channel dimension, and further integrated with 1 × 1 Conv to obtain enhanced features.

In the decoder, firstly, the intermediate features extracted by ComNet with 4× downsampling are integrated by using 1 × 1 Conv. Then, the enhanced features output from ASPP are 4× upsampled and concatenated with the intermediate features in channel dimension. Then, the features are further integrated by using 3 × 3 Conv. Finally, the background filtering results of aeroengine blades are obtained via 4× upsampling.

### 3.2. Three-Dimensional Reconstitution

**Figure 3:**
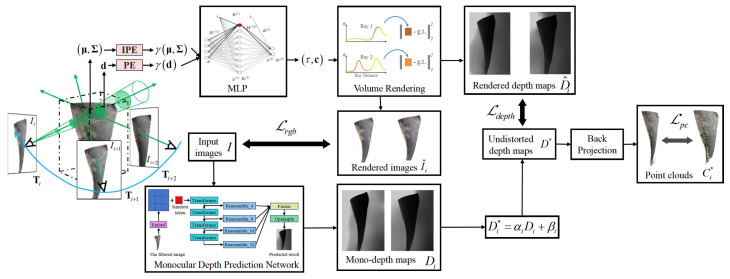
Overview of the implicit 3D reconstruction method based on cone sampling and “pose–NeRF” joint optimization.

To solve the problems of over-blurring, aliasing artifacts, and other problems caused by smooth surface, weak texture information, and large changes in the relative depth of field, as well as the problem that it is difficult to precisely control the pose of the borescope lens due to the limitations of the probe hole and the internal complex structure of aeroengine, an implicit 3D reconstruction method based on cone sampling and “pose–NeRF” joint optimization is adopted in this section. Our method takes a sequence of aeroengine blade images as input. Firstly, the mono-depth map is obtained through the monocular depth prediction network, and the blade point cloud is obtained according to the mono-depth map. At the same time, the rendered depth maps and rendered images of the blades are obtained through the cone sampling based NeRF. Then, the camera pose and NeRF are jointly optimized by computing the loss between the real images and the rendered images, the mono-depth maps and the rendered depth maps, and the point cloud between two adjacent frames. The pipeline of our implicit 3D reconstruction method is shown in Figure 3.

#### 3.2.1. Monocular Depth Prediction Network

**Figure 4:**
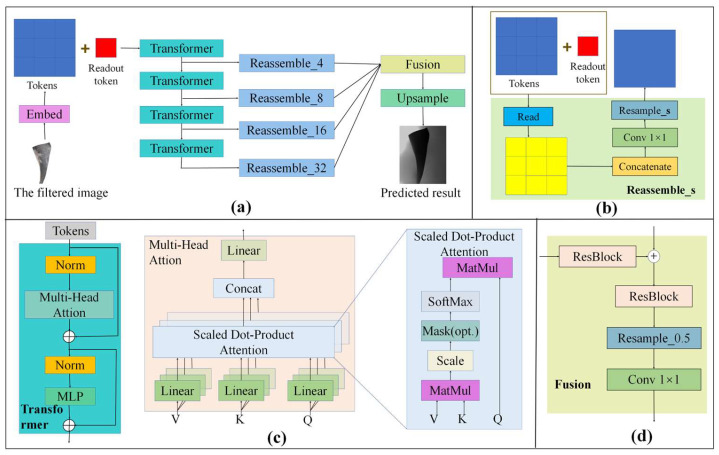
Pipeline of monocular depth prediction network. (**a**) Overall framework of the network. (**b**) The architecture of the reassemble module. (**c**) The illustration of the transformer. (**d**) The architecture of the fusion module.

The pipeline of the monocular depth prediction network is shown in Figure 4. The input image is divided into non-overlapping patches, and then the features of the patches are integrated and extracted by ResNet-50 [42] to obtain the feature units. In this paper, we refer to these feature units as tokens. Then, the tokens are embedded in position to preserve their position information. Like ViT [48], a patch-independent readout token is added. A transformer module is used to further extract features and perform downsampling. Then, the reassemble module is used to integrate the features extracted from different stages into image-like representations with different resolutions. Finally, a fusion module is used to fuse features with different levels of semantic information and perform upsampling to obtain the predicted results.

The input image *X* with size *H × W* is divided into *N_p_* patches *P* ∈ *R^p×p^*, where *N_p_* = *HW/P*^2^, and then integrated through ResNet-50 to obtain tokens. A patch-independent readout token is added to obtain the final tokens *T =* [*t*_0_, *t*_1_, *···t_Np_*]*^T^*∈*R^(Np+^*^1*)×dt*^. The transformer module is composed of a normalization layer (Norm), a multi-head attention block, and a multilayer perceptron (MLP). The output feature $F_{Tout}^{i}={\left[f_{Tout}^{0},f_{Tout}^{1}\ldots f_{Tout}^{N_{p}}\right]}^{T}\in R^{(N_{p}+1)\;\times \;d_{t}}$ of the *i*th transformer can be computed by Equation (3), where $F_{\mathrm{Tin}}^{i}$ ∈ *R^(Np+^*^1*) × dt*^ is the input feature of the *i*th transformer, *Norm*(•) is the normalization layer, *MLP*(•) is the multilayer perceptron, and *MHA*(•) is multi-head attention. In this study, *i* ∈ [1, 4].

(3) $$\begin{array}{l} \left\{\begin{array}{l} F_{\mathrm{Tout}}^{i}=MLP(Norm(MHA(Norm(F_{\mathrm{Tin}}^{i}))+F_{\mathrm{Tin}}^{i}))+MHA(Norm(F_{\mathrm{Tin}}^{i}))+F_{\mathrm{Tin}}^{i}\\ F_{\mathrm{Tin}}^{i}=\left\{\begin{array}{c} T,i=1\\ F_{\mathrm{Tout}}^{i-1},i>1 \end{array}\right. \end{array}\right. \end{array}$$

As shown in Figure 4c, the multi-head attention module is a stack of multiple scaled dot-product attention. Multi-head attention allows the model to jointly attend to information from different representation subspaces at different positions. With a single attention head, averaging inhibits this. The output features *F_Mout_* ∈ *R^(Np+^*^1*) ×dt *^ of multi-head attention can be computed by Equation (4), where *Concat* is concatenation, *F_in_* ∈ *R^(Np+^*^1*)×dt*^ is the input features of scaled dot-product attention, *F_Sout_* ∈ *R^(Np+^*^1*)×dt*^ is the output features of scaled dot-product attention, *Q*,*K*,*V* ∈ *R^dt×dt^* are the respective projection matrices for queries, keys and values, and *W* ∈ *R^hdt×dt^* is the integrating projection matrices.

(4) FMout=Concat(FSout1,FSout2,…,FSouth)W FSouti=softmax((FinQi)(FFinKi)Tdt)(FinVi) 

The output features $F_{\mathrm{Tout}}^{i}$ of any transformer are reassembled into image-like representations at different resolutions by the reassemble module. As shown in Figure 4b, the reassemble module consists of three stages: read, concatenate, and resample. The read block maps the readout token into other tokens: $R^{(N_{p}+1)\times d_{t}}\to R^{N_{p}\times d_{t}}$. In the read block, the feature $f_{\mathrm{Tout}}^{0}\in R^{d_{t}}$ corresponding to the readout toke $t_{0}\;\in \;R^{d_{t}}$ is concatenated to $f_{\mathrm{Tout}}^{j}\in R^{d_{t}}$, *j* ∈ [1,*N_p_*], and then the features are integrated by the MLP.

The output features *F_Read_*∈$R^{N_{p}\times d_{t}}$ of the read module can be computed as follows: $F_{\mathrm{Read}}$ = $\left\{MLP(Concat(f_{\mathrm{Tout}}^{1},f_{\mathrm{Tout}}^{0})),\ldots ,MLP(Concat(f_{\mathrm{Tout}}^{N_{p}},f_{\mathrm{Tout}}^{0}))\right\}$, where $f_{\mathrm{Tout}}^{j}$ is the jth vector of $F_{\mathrm{Tout}}^{i}$. Then, a concatenate block is used to stack the features *F_Read_* into image-like representations, resulting in a feature map *F_Concat_* ∈ $R^{\frac{H}{p}\times \frac{W}{p}\times D}$ with size H/p×W/p and channel *d_t_*:$R^{N_{p}\times D}\to R^{\frac{H}{p}\times \frac{W}{p}\times D}$. In the resample block, the feature map *F_Concat_* is integrated by 1 × 1 Conv. Then, if *s* ≥ *p*, the feature map is downsampled by 3 × 3 Conv, otherwise, the feature map is upsampled by 3 × 3 transpose convolution. Finally, the reassemble features *F_Reassem_*∈RHs×Ws×D^ are obtained, where *s* is the sampling multiple. In the fusion module, the feature maps with different resolutions extracted by transformers are fused to obtain features with richer semantic information. As shown in Figure 4d, the fusion module consists of two ResBlocks, a resample block, and a 1 × 1 Conv.

#### 3.2.2. Cone Sampling Based NeRF

**Figure 5:**
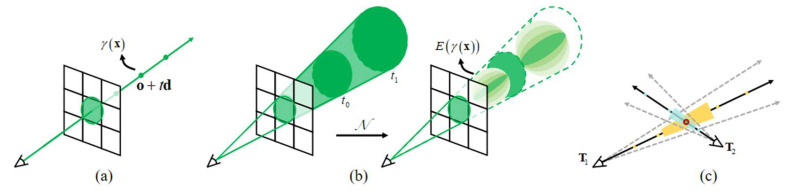
Spatial sampling. (**a**) Ray sampling. (**b**) Cone sampling. (**c**) Illustration of comparison between ray sampling and cone sampling.

NeRF [12] proposed by Mildenhall et al. is an implicit 3D reconstruction method, opening up a new direction for the field of 3D reconstruction. However, the ray-sampling method of NeRF was proposed for a general dataset with strong texture information and complex surface changes, and requires the camera poses to be roughly distributed on a hemisphere around the target. However, aeroengine blades have the characteristics of smooth surface and weak texture information. And due to the limitations of aeroengine probe holes and complex internal structures, the borescope lens cannot be controlled on the hemisphere around blades, resulting in an inconsistent distance between the lens and the blade, and causing changes in the scale of the images. These factors cause issues such as excessive blurring and aliasing artifacts in 3D reconstruction. To address these issues, an improved NeRF method based on cone sampling, as shown in Figure 5a, is introduced.

As is well-known, the color of a pixel is the integration of all incident light within the pixel cone. However, the existing NeRF methods consider it to be a ray and set sampling points on the ray, as shown in Figure 5b. Figure 5c shows that these sampling points ignore the shape and size of the volume viewed by each ray, so two different cameras imaging the same position at different scales may produce the same ambiguous point-sampled feature. The method adopted in this paper improves this problem by projecting a cone from the camera. Instead of constructing positional encoding (PE) features from an infinitesimally small point in space, we construct an integrated positional encoding (IPE) representation of the volume covered by each conical frustum (simplified to a trapezoid in Figure 5c). These changes allow the MLP to reason about the size and shape of each conical frustum, instead of just its centroid. To encode the position of conical frustums, we assume that the points inside conical frustums conform to the spatial Gaussian distribution. Then, IPE is performed on the expectation *µ* and covariance *Σ* of the spatial Gaussian distribution, as shown in Equation (5). The PE of direction still adopts the encoding method in [12].

(5) $$\begin{array}{l} \left\{\begin{array}{l} \gamma (\mu ,\Sigma )=E_{x∼N(\mu_{\gamma },\Sigma_{\gamma })}[\gamma (x)]=\left[\begin{array}{c} sin(\mu_{\gamma })\circ exp(-(1/2)diag(\Sigma_{\gamma }))\\ cos(\mu_{\gamma })\circ exp(-(1/2)diag(\Sigma_{\gamma })) \end{array}\right]\\ P=\left[\begin{array}{lll} 1\;0\;0\;2\;0\;0\; & & 2^{L-1}\;\;0\;\;\;\;\;0\\ 0\;1\;0\;0\;2\;0 & \ldots & 0\;\;\;\;\;2^{L-1}\;\;0\\ 0\;0\;1\;0\;0\;2\; & & 0\;\;\;\;\;0\;\;\;\;\;\;2^{L-1} \end{array}\right]\\ \gamma (x)=\left[\begin{array}{c} sin(Px)\\ cos(Px) \end{array}\right]\\ \Sigma_{\gamma }=P\Sigma P^{T}\\ \mu_{\gamma }=P\mu \end{array}\right. \end{array}$$

The IPE of each conical frustum *γ* (*µ*, Σ) and the PE of direction *γ* (*d*) are provided as input to an MLP parameterized by weights *Θ*, which outputs a density *τ* and an RGB color *c*: $\left[\tau_{k},c_{k}]=MLP(\gamma (\mu_{k},\Sigma_{k}),\gamma (d_{k});\Theta \right)$. Then, the classic volume rendering theory is used to render the colors of all cones passing through the scene. As shown in Equation (6), the expected color *C(r)* on the pixel is obtained by integrating on the central ray *r(t) = o + td* of the cone, where $T(t)=exp(-\int_{t_{n}}^{t}\tau (r(s))ds)$ denotes the accumulated transmittance along the ray from *t_n_* to *t*.

(6) $$C(r)=\int_{t_{n}}^{t_{f}}T(t)\tau (r(t))c(r(t),d)dt$$

#### 3.2.3. Joint Optimization Loss Function

Given *N* images $I=\{I_{i}|i=1,2,\ldots N\}$ with their camera poses $P=\{P_{i}|i=1,2,\ldots N\}$, NeRF can be optimized by minimizing photometric loss Lrgb=∑i=1N∥Ii−I^i∥22 between rendered images I^={I^i∣i=1,2,…N} and captured images I:

(7) Θ*=arg minΘLrgb(I^∣I,P)

The key to the joint optimization of camera pose and NeRF is to adjust the projection of camera light onto the variable camera pose P, because camera light *r* is a function of camera pose. Mathematically, this joint optimization can be formulated as Equation (8), where P^={P^i∣i=1,2,…N} denotes camera poses that are updated during optimizing. Camera pose $P_{i}$ for frame $I_{i}$ is a transformation $T_{i}=[R_{i}|t_{i}]$ with a rotation $R_{i}\in SO(3)$ and a translation $t_{i}\in R^{3}$.

(8) Θ*,P*=arg minΘLigb(I^,P^∣I)

The mono-depth sequence $D=\{D_{i}|i=1,2,\ldots N\}$ of aeroengine blades is obtained through the monocular deep prediction network. However, the obtained mono-depth maps are distorted and do not have multi-view consistency. To generate high-quality point clouds and further optimize the relative camera pose through point clouds, restoring the multi-view consistency of mono-depth maps is necessary. Therefore, we introduce a sequence of destruction parameters $\Psi =\{(\alpha_{i},\beta_{i})|i=1,2,\ldots N\}$ for all frames, where $\alpha_{i}$ and $\beta_{i}$ denote a scale and a shift factor. Then, the undistorted depth map $D_{i}^{*}$ with multi-view consistency is defined as follows:

(9) $$D_{i}^{*}=\alpha_{i}D_{i}+\beta_{i}$$

The multi-view consistent depth map $D_{i}^{*}$ is obtained through joint optimization of $\alpha_{i}$ and $\beta_{i}$. Depth loss $L_{depth}$ to optimize $\alpha_{i}$ and $\beta_{i}$ by computing the difference between the undistorted depth map $D_{i}^{*}$ and the NeRF rendered depth map D^i is designed as follows:

(10) Ldepth=∑i=1N∥Di*−D^i∥

To further optimize the camera pose, point cloud loss $L_{pc}$ is proposed. The loss continuously optimizes the camera pose by computing the chamfer distance $l_{cd}\left(C_{i}^{*},C_{j}^{*}\right)$ between different frames point clouds, rather than independently optimizing each camera pose. Point cloud loss alleviates the problem of incorrect camera pose caused by NeRF overfitting the target image. We back-project the undistorted depth maps $D^{*}=\{D_{i}|i=1,2,\ldots N\}$ to obtain point cloud $C^{*}=\{C_{i}^{*}|i=1,2,\ldots N\}$, and then optimize the relative pose between cameras in different frames by minimizing point cloud loss $L_{pc}$, which is shown in Equation (11):

(11) $$\left\{\begin{array}{l} L_{pc}=\sum_{\left(i,j\right)}l_{cd}\left(C_{j}^{*},T_{ji}C_{i}^{*}\right)\\ l_{cd}\left(C_{i}^{*},C_{j}^{*}\right)\underset{c_{i}^{*}\in C_{i}^{*}}{\sum }\underset{c_{j}}{min}c_{C_{j}^{*}}^{*}{∥c_{i}^{*}-c_{j}^{*}∥}_{2}+\underset{c_{j}\in C_{j}^{*}}{\sum }\underset{c_{i}\in C_{i}^{c_{i}}}{min}{∥c_{i}^{*}-c_{j}^{*}∥}_{2} \end{array}\right.$$

where $T_{ji}=T_{j}T_{i}^{-1}$ represents the related pose that transforms point cloud $C_{i}^{*}$ to $C_{j}^{*},(i,j)$ denotes indices of a consecutive pair of instances. The overall loss function is shown in Equation (12), where $\lambda_{1}$, $\lambda_{2}$ are the weighting factors for respective loss terms.

(12) $$L=L_{rgb}+\lambda_{1}L_{depth}+\lambda_{2}L_{pc}$$

## 4. Experiments and Results

In this section, experiments are carried out to research the performance of the method for the in situ high-fidelity 3D reconstruction of aeroengine blades proposed in this paper. The experiments include background filtering experiments (Section 4.2) and 3D reconstruction experiments (Section 4.3). In background filtering experiments, we study the performance of ComBFNet for aeroengine blades. In 3D reconstruction experiments, we study the performance of a cone sampling-based “pose–NeRF” joint optimization implicit 3D reconstruction model in this paper.

### 4.1. Implementation Details

#### 4.1.1. Experimental Conditions

Table 1 shows the training environment and some parameter settings for the experiments. The experiments are conducted on a server with NVIDIA GeForce RTX3080 GPU. We implement our model in PyTorch. The Adam [49] optimization method is adopted in background filtering experiments, and SGD [50] optimization method is adopted in 3D reconstruction experiments. The initial learning rate is set to 10^−4^, and cosine annealing with warm restart is used to adjust the learning rate [51]. The background filtering model is trained for 200 epochs, with an average training time of 14 h, and the implicit 3D reconstruction model is trained for 1 × 10^5^ iters, with an average training time of 10.5 h.

#### 4.1.2. Dataset

**Figure 6:**
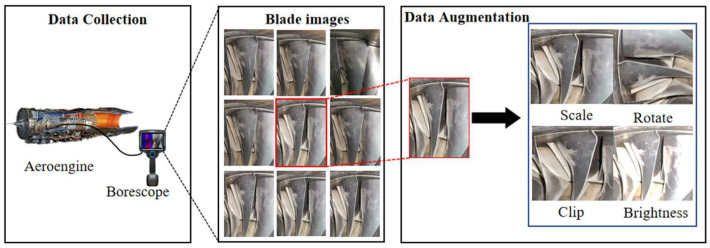
The construction of ABB dataset.

As shown in Figure 6, an industrial borescope is used to capture sequential images of aeroengine blades. Each blade is photographed, with 40 images captured from different angles to obtain an aeroengine blades borescope dataset (ABB dataset). In the background filtering experiments, to enhance the generalization and robustness of our model, random scaling, cropping, rotation, and brightness change are used to augment the dataset. These data augmentation methods can reduce the sensitivity of the model to blade position, size, angle, and ambient light, and improve the background filtering performance of the model. In the 3D reconstruction experiments, 40 images of each blade taken from different angles can be used as an independent dataset to train the implicit 3D reconstruction model.

#### 4.1.3. Metrics

The mean intersection over union (*mIoU*) is used to evaluate the background filtering model. As shown in Equation (13), *mIoU* is the average of the ratio of the intersection and union of the true and predictive values of all targets.

(13) $$mIOU=\frac{1}{k+1}\sum_{i=0}^{k}\frac{TP}{FN+FP+TP}$$

where *k* is the total number of target categories, *TP* is true positives, indicating that both the true and predictive value are positive, *FP* is false positives, indicating that the true value is negative and the predictive value is positive, and *FN* is false negatives, indicating that the true value is positive and the predictive value is negative.

We evaluate the implicit 3D reconstruction model using three metrics: (1) peak signal-to-noise ratio (*PSNR*) [19], the ratio of maximum signal power to signal noise power; (2) structural similarity (*SSIM*) [52], an indicator that measures the similarity between two images; and (3) learned perceptual image patch similarity (*LPIPS*) [19], used to measure the difference between two images, which is more in line with human perception than traditional methods.

(14) PSNR=10×log10MAXI2MSESSIMx,y=2μxμy+c12σxy+c2μx2+μy2+c1σx2+σy2+c2LPIPSx,y=∑l1HlWl∑h,w∥wl⊙(z^xhwl−z^yhwl)∥22

In PSNR, mean-square error $MSE=\frac{1}{mn}\sum_{i=0}^{m-1}\sum_{j=0}^{n-1}[I(i,j)-K(i,j){]}^{2}$ is the difference between clean image *I* and noisy image *K* of size m × n, and *MAX_I_* is the maximum possible pixel value. SSIM mainly considers three key features of images *x* and *y*: brightness, contrast, and structure, where $\mu_{x}=\frac{1}{N}\sum_{i=1}^{N}x_{i}$ and $\mu_{y}=\frac{1}{N}\sum_{i=1}^{N}y_{i}$ are the average grayscale, $\sigma_{x}={\left(\frac{1}{N-1}\sum_{i=1}^{N}{\left(x_{i}-\mu_{x}\right)}^{2}\right)}^{\frac{1}{2}}$ and $\sigma_{y}={\left(\frac{1}{N-1}\sum_{i=1}^{N}{\left(y_{i}-\mu_{y}\right)}^{2}\right)}^{\frac{1}{2}}$ are the grayscale standard deviation, $\sigma_{xy}=\frac{1}{N-1}\sum_{i=1}^{N}(x_{i}-\mu_{x})(y_{i}-\mu_{y})$ is the grayscale covariance, and *L* is the range of pixel values, $c_{1}={\left(k_{1}\cdot L\right)}^{2}$, $c_{2}={\left(k_{2}\cdot L\right)}^{2}$, where *k*_1_ = 0.01, and *k*_2_ = 0.03. In LPIPS, *H* and *W* are the height and width of image, *w* is the weight, and *z* is the feature map after activation and normalization.

Because the method proposed in this paper is an implicit reconstruction method, explicit point cloud data cannot be directly obtained after training. Therefore, to evaluate the geometric accuracy of the reconstruction, we used the CLI of NeRFStudioto to carry out density threshold sampling to convert the implicit volume density into an explicit point cloud. At the same time, we collected the point cloud data of aeroengine blades using a scanner under non-in situ conditions and applied it as the ground truth. Finally, the reconstructed point cloud was compared with the scanned point cloud in CloudCompare 2.13. Although 3D reconstruction is increasingly widely applied, there is currently a lack of quality evaluation methods based on specified standards or criteria. By reviewing the literature [53] and the CloudCompare documentation, we adopted standard deviation (*STD*) and root mean square error (*RMSE*) for evaluation:

(15) $$\left\{\begin{array}{c} STD=\sqrt{\frac{1}{N-1}\sum_{j=1}^{N}{\left(X_{j}-\underline{X}\right)}^{2}}\\ RMSE=\sqrt{\frac{\sum_{j=1}^{N}\left|X_{j}\right|}{N}}\;\;\;\;\;\;\;\;\;\;\;\;\;\;\;\;\;\;\; \end{array}\right.$$

Among them, *N* represents the number of observed point clouds, *X_j_* is the closest distance between each point and the corresponding reference point or surface, and $\underline{X}$ represents the average observed distance.

### 4.2. Background Filtering Experiments

**Figure 7:**
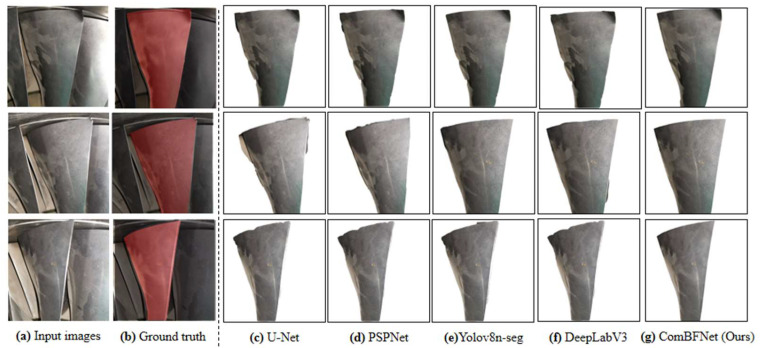
Qualitative visualization background filtering results of different methods on ABB dataset.

To verify the performance of the background filtering model for aeroengine blades designed in this paper, we conduct comparative experiments on ABB dataset to study the performance differences between our model and the existing semantic segmentation models. The baselines used for comparison are U-Net [54], PSPNet [55], Yolov8n-seg and DeepLabV3 [56]. In addition, we also validated the effectiveness of the backbone ComNet designed in this paper by conducting ablation experiments. The models are trained with 200 epochs, and the experimental environment and parameter settings are completely consistent. The results are shown in Table 2, and the qualitative visualization results are shown in Figure 7. The evaluation metric is *mIoU* (%).

Table 2 indicates that ComBFNet achieves the best background filtering results on the ABB dataset, proving the feasibility of our model. This is mainly due to the innovative backbone designed based on complex network theory in this paper. The comparison of different backbones in the experiment not only verifies the effectiveness of feature extraction in ComNet, but also proves the correctness of our idea of designing neural network architecture based on complex network theory. Whether it is human visual neural networks, transportation networks, information transmission networks, etc., these are all complex networks between regular networks and random networks. Therefore, the backbone for extracting image features designed as a complex network is in line with objective laws. ComNet allows feature flows to be transmitted in different paths, so that the high-level semantic features extracted by more convolution layers and the low-level semantic features extracted by fewer convolution layers can be adaptively fused to obtain features with richer semantic information, which significantly improves the feature extraction ability of the network.

### 4.3. Three-Dimensional Reconstruction Experiments

In this section, we conduct experiments to verify the feasibility and effectiveness of the in situ high-fidelity 3D reconstruction method for aeroengine blades proposed in this paper. Ablation experiments are also conducted to study the effects of background filtering, cone sampling and joint optimization loss function on the 3D reconstruction method. In this paper, sequential frame images (including 40 frames) taken from different angles of each blade in the ABB dataset are used as an independent dataset to train the model.

#### 4.3.1. Comparison Experiments

In this section, Instant-NGP [13], NeuS [16], Mip-NeRF [14], and Point-NeRF [17] are selected as baselines to carry out comparison experiments to study the reconstruction performance of the PoseNeRF method for the in situ 3D reconstruction of blades proposed in this paper, where woBF is without background filtering and wBF is with background filtering. Incremental SFM [20] is used to pre-estimate the camera pose in the baseline methods. All models are trained 1 × 10^5^ iters. *PSNR*, *SSIM* and *LPIPS* are used as the measurement indicators. The larger the *PSNR* and *SSIM*, the smaller the *LPIPS*, indicating the better performance of the reconstruction method.

The results are shown in Table 3. The performance of our proposed in situ high-fidelity 3D reconstruction method for aeroengine blades is superior to the other five methods, with *PSNR*, *SSIM*, and *LPIPS* reaching 25.61, 0.749, and 0.212, respectively. This is because the “pose–NeRF” joint optimization method is adopted to realize the end-to-end optimization of camera pose and NeRF during the 3D reconstruction process, instead of using SFM to pre-estimate the camera pose separately. This method avoids the cumulative error in NeRF optimization caused by pose pre-estimation. In NeRF, the ray sampling method is changed to cone sampling, and IPE based on the characteristics of conicla sampling is proposed. Cone sampling effectively improves the problems of excessive blurring and aliasing artifacts caused by smooth blade surfaces, weak texture information, and large camera pose changes in 3D reconstruction, significantly improving the fidelity of blade 3D reconstruction. The background filtering model based on complex network theory is utilized to filter out useless factors from 2D images, avoiding interference on blade 3D reconstruction.

**Figure 8:**
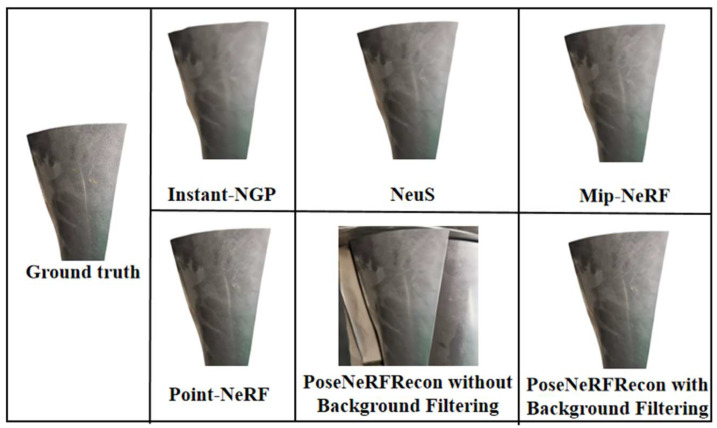
Qualitative visualization 3D reconstruction results of different methods on ABB dataset.

The qualitative visualization results of the 3D reconstruction of the blades are shown in Figure 8. Compared to other methods, our method reconstructs the blades more clearly. In addition, the results prove that background filtering has a positive effect on improving the fidelity of the 3D reconstruction of aeroengine blades.

Table 4 shows the *STD* and *RMSE* results between the reconstructed point cloud and the scanned point cloud, which can be used to evaluate the geometric accuracy of the reconstruction model. Similarly, we compare the model proposed in this paper with the four representative models mentioned earlier. Without background filtering, compared to Instant NGP, Mip NeRF, Point NeRF, and NeuS, the *RMSE* and *STD* of the reconstructed point cloud from our model are slightly higher than those of Instant-NGP but significantly outperform the other three models. After introducing background filtering, the *RMSE* and *STD* values were 0.69 mm and 0.47 mm, respectively, further reducing by 16.87% and 22.95%. Therefore, the experimental results show that our model method has the lowest geometric error, which demonstrates that the “pose–NeRF” joint optimization scheme combined with background filtering can significantly improve the reconstruction accuracy of aircraft engine blades.

#### 4.3.2. Ablation Experiments

In Section 4.3.1, the effectiveness of the in situ high-fidelity 3D reconstruction method for aeroengine blades is verified, but the impact of the key modules proposed in this paper on the 3D reconstruction method and the interaction relationships between the modules are not studied. In this section, we conduct ablation experiments to study the effects of background filtering, cone sampling, and joint optimization loss function on 3D reconstruction methods. For the study of cone sampling, ray sampling is considered as an alternative. For loss function, we explore the impact of depth loss $L_{depth}$ and point cloud loss $L_{pc}$ on 3D reconstruction by changing the weighting factors $\lambda_{1}$ and $\lambda_{2}$.

The results of ablation experiment are shown in Table 5. To explore the impact mechanism of various key modules on the proposed 3D reconstruction method, we also introduce relative pose error (RPE) [15,57,58,59] as the metric for camera pose optimization in this paper. RPE measures the relative pose errors between pairs of images, which are the consistency of relative rotation error (RPEr) and the relative translation error (RPEt). Where Cone is cone sampling, Ray is ray sampling, woBF is without background filtering, and wBF is with background filtering.

Table 5 indicates that background filtering, cone sampling, depth loss, and point cloud loss all improve the 3D reconstruction performance of the model. Among them, cone sampling has the greatest improvement in 3D reconstruction. This is because cone sampling is more in line with the objective situation of volume rendering and avoids the problem of ray sampling ignoring the shape and size of spatial volume, which results in two different cameras imaging the same position at different scales and may produce the same ambiguous point-sampled feature. The joint optimization loss function composed of photographic loss, depth loss, and point cloud loss also improves the performance of the 3D reconstruction model. With the increase in $\lambda_{1}$ and $\lambda_{2}$, the fidelity of the 3D reconstruction of the blades gradually improves, because depth loss and point cloud loss have a greater effect on 3D reconstruction. In addition, Table 4 reveals that the changes in RPEr and RPEt are mainly influenced by $\lambda_{1}$ and $\lambda_{2}$, confirming that the joint optimization loss function mainly improves the fidelity of the 3D reconstruction of the blades by optimizing the camera pose.

## 5. Conclusions

In this paper, a NeRF-based in situ high-fidelity 3D reconstruction method for aeroengine blades is proposed for the digital twin for the real-time health management and monitoring of aircraft engines. We analyze and summarize the existing issues of current ex situ 3D reconstruction methods and the reasons why existing image-based 3D reconstruction methods (MVS and NeRF) cannot be directly used for in situ 3D reconstruction of aeroengine blades. To achieve an in situ high-fidelity 3D reconstruction of aeroengine blades, ComBFNet, based on complex network theory, is designed to filter out useless background information in the 2D images of blades collected by the borescope. Then, a “pose–NeRF” joint optimization 3D reconstruction method based on cone sampling is adopted to achieve in situ 3D reconstruction. The method solves the challenges of 3D reconstruction of blades caused by smooth surface, weak texture information and difficulty in the accurate control of the borescope lens.

Extensive experiments are conducted on the general dataset and the ABB dataset to verify the effectiveness and progressiveness of our method. Background filtering experiments show that the background filtering effect of ComBFNet is better than other general models, and its *mIoU* reaches 95.7%. The experiment also verifies the effectiveness of the backbone designed based on complex network theory. The 3D reconstruction experiment confirms the performance of the adopted “pose–NeRF” joint optimization 3D reconstruction method based on cone sampling. The ablation experiment results also indicate that the joint optimization loss function can improve the 3D reconstruction performance of the model mainly by affecting the optimization of camera pose. This is an attempt at an in situ reconstruction of aeroengine blades, and we hope that the proposed method can bring illuminating thinking to the research of digital twins in aeroengines.

## Figures and Tables

**Table 1:** The condition and parameter settings of the experiments.

Experimental Condition	Background Filtering	3D Reconstruction
Operating system	Windows 10	Ubuntu 20.04
Graphics card	GeForce RTX 3080	GeForce RTX 3080
PyTorch	1.7.0	1.8.0
Optimization	Adam	SGD
batch_size	8	8

**Table 2:** The results of background filtering experiments.

Model	Backbone	mIoU (%)
U-Net	ResNet50	85.9
	ComNet	88.8
PSPNet	ResNet50	87.1
	ComNet	90.2
Yolov8n-seg	Xception	91.8
	ComNet	93.1
DeepLabv3	ResNet50	93.4
	ComNet	94.5
ComBFNet (ours)	ComNet	95.5

**Table 3:** The results of 3D reconstruction comparison experiments.

Method	PSNR	SSIM	LPIPS
Instant-NGP	21.94	0.618	0.367
NeuS	22.92	0.655	0.357
Mip-NeRF	23.57	0.672	0.296
Point-NeRF	24.68	0.689	0.275
PoseNeRF woBF	24.53	0.687	0.278
PoseNeRF wBF	25.59	0.719	0.239

**Table 4:** The geometric accuracy results of 3D reconstruction comparison experiments.

Method	STD	RMSE
Instant-NGP	0.57	0.78
NeuS	1.95	2.16
Mip-NeRF	1.46	1.24
Point-NeRF	1.72	1.65
PoseNeRF woBF	0.61	0.83
PoseNeRF wBF	0.47	0.69

**Table 5:** The results of 3D reconstruction ablation experiments.

	Cone	Ray	wBF	woBF	PSNR	SSIM	LPIPS	RPEr	RPEt
Ray + woBF + (y1 = 0.2, y2 = 0.2)		√		√	20.08	0.549	0.445	0.329	0.087
Cone + woBF + (y1 = 0.2, y2 = 0.2)	√			√	22.38	0.619	0.279	0.229	0.083
Ray + wBF + (y1= = 0.2, y2 = 0.2)		√	√		20.59	0.580	0.391	0.339	0.094
Ray + woBF + (y1 = 0.6, y2 = 0.6)		√		√	22.01	0.619	0.331	0.231	0.078
Ray + woBF + (y1 = 1.0, y2 = 1.0)		√		√	22.34	0.629	0.299	0.174	0.069
Ray + wBF + (y1 = 1.0, y2 = 1.0)		√	√		23.29	0.631	0.319	0.175	0.071
Cone + woBF + (y1 = 1.0, y2 = 1.0)	√		√		24.13	0.687	0.268	0.148	0.064
Cone + wBF + (y1 = 0.2, y2 = 0.2)	√	√			23.45	0.659	0.321	0.314	0.085
Cone + wBF + (y1 = 0.6, y2 = 0.6)	√	√			24.48	0.702	0.251	0.212	0.075
Cone + wBF + (y1 = 1.0, y2 = 1.0)	√	√			25.59	0.719	0.239	0.147	0.068

## Data Availability

The datasets generated and/or analyzed during the current study are available from the corresponding author on reasonable request.
